# A New Method of Trephining the Skull

**Published:** 1882-04

**Authors:** John B. Roberts


### A NEW METHOD OF TREPHINING THE SKULL AND OTHER BONES.

A short time ago I became cognizant of the method used by Prof. James E. Garretson for the removal of the coccyx. This he effects by uncovering the bone and grinding it away with the Bonwill surgical engine, armed with a burr. A few days later, by his invitation, I saw him remove in a similar manner the right superior maxilla, which was the seat of an antral exostosis. The delicacy of manipulation, the absence of facial scarring, and the undoubted power of the engine, combined to give me a very high appreciation of its possibilities. Especially was this the case because my experience some three years ago with the so-called dental engine was very unsatisfactory in surgical operations on bone.

During Dr. Garretson's operation some one of the bystanders suggested to me that the engine might be used for trephining, and, as I had shortly before been teaching this operation to my class, I was struck with the idea. It has been heretofore suggested, I believe, that the engine might be employed to drive a trephine, and thus cut out a disk or button of bone.

My idea, however, was that, as the ordinary trephines are usually of too great diameter, and cause larger openings than are required for the insertion of the elevator, it would be practicable to bore a small hole in the skull by using in the engine a burr cut or roughened on its flat extremity.

As no patient was at hand, I utilized a cadaver for the experimental demonstration, and fractured the skull by means of a hatchet. I found that the burr called by the dental instrument-makers a fissure-burr, and which has a cut face, answered admirably. I applied it to the sound bone at the edge of the depressed fracture, and found that I could quite readily make a circular cavity in the outer table. This was carefully deepened until the vitreous table was perforated. As there was no disk to remove, and as the burr, which I kept moistened with water dropping from a cloth, threw out all bone-dust, the depth and character of the perforation were readily watched. When the skull was thus pierced by a round orifice about one-quarter of an inch in diameter, the elevator was inserted and the depressed fragments elevated, and, where loose, removed. Sharp and irregular edges were equally well trimmed smooth, or cut away by the burr.

When the rapidly rotating burr is placed in contact with soft tissues, as one's finger, it can be pressed upon with considerable firmness without abrading the surface, while osseous tissue is quickly ground away. Hence, it seems as if the meninges of the brain might be touched by the burr without injury being inflicted at the time the vitreous table is perforated. In fact, I am inclined to believe that the dura mater would be pushed in front of the burr and remain practically uninjured. This can be tested only in living animals or human beings, because in the cadaver the brain does not entirely fill the cranial cavity, though the dura mater may remain attached to the inner table. The depressed fracture, moreover, usually pushes the dura matter downward, which would thus be likely to be torn off from the sound bone nearest the depression. —*John B. Roberts, M.D., in Philadelphia Med. Times.*

